# Dextransucrase Expression Is Concomitant with that of Replication and Maintenance Functions of the pMN1 Plasmid in *Lactobacillus sakei* MN1

**DOI:** 10.3389/fmicb.2017.02281

**Published:** 2017-11-21

**Authors:** Montserrat Nácher-Vázquez, José A. Ruiz-Masó, María L. Mohedano, Gloria del Solar, Rosa Aznar, Paloma López

**Affiliations:** ^1^Department of Molecular Microbiology and Infection Biology, Biological Research Center, Spanish National Research Council (CSIC), Madrid, Spain; ^2^Department of Food Safety and Preservation, Institute of Agrochemistry and Food Technology, CSIC, Paterna, Spain; ^3^Department of Microbiology and Ecology, University of Valencia, Burjassot, Spain

**Keywords:** dextran, dextransucrase, lactic acid bacteria, *Lactobacillus sakei*, plasmid, probiotics

## Abstract

The exopolysaccharide synthesized by *Lactobacillus sakei* MN1 is a dextran with antiviral and immunomodulatory properties of potential utility in aquaculture. In this work we have investigated the genetic basis of dextran production by this bacterium. Southern blot hybridization experiments demonstrated the plasmidic location of the *dsrLS* gene, which encodes the dextransucrase involved in dextran synthesis. DNA sequencing of the 11,126 kbp plasmid (pMN1) revealed that it belongs to a family which replicates by the theta mechanism, whose prototype is pUCL287. The plasmid comprises the origin of replication, *repA, repB*, and *dsrLS* genes, as well as seven open reading frames of uncharacterized function. *Lb. sakei* MN1 produces dextran when sucrose, but not glucose, is present in the growth medium. Therefore, plasmid copy number and stability, as well as *dsrLS* expression, were investigated in cultures grown in the presence of either sucrose or glucose. The results revealed that pMN1 is a stable low-copy-number plasmid in both conditions. Gene expression studies showed that *dsrLS* is constitutively expressed, irrespective of the carbon source present in the medium. Moreover, *dsrLS* is expressed from a monocistronic transcript as well as from a polycistronic *repA-repB-orf1-dsrLS* mRNA. To our knowledge, this is the first report of a plasmid-borne dextransucrase-encoding gene, as well as the first time that co-transcription of genes involved in plasmid maintenance and replication with a gene encoding an enzyme has been established.

## Introduction

Lactic acid bacteria (LAB) play an important role in the production of fermented foods based on milk, meat, and vegetables as well as alcoholic beverages, due to their metabolic pathways, whose products contribute to food safety (e.g., lactic acid or hydrogen peroxide) and to the organoleptic characteristics [e.g., the diacetyl and other aroma compounds or texturizing exopolysaccharides (EPS)]. In addition, some LAB have beneficial health characteristics (probiotic properties) or metabolic capacities such as the production of enzymes (amylases, phytases), vitamins (folates, riboflavin), or EPS, which are of particular interest for the agro-food industry and for the formulation of new functional foods (Anastasio et al., 2010; Badel et al., 2011; Capozzi et al., 2012).

The EPS produced by LAB can be classified by (i) composition, because they include different types of bonds and monosaccharide subunits; (ii) types and degrees of branching; (iii) molecular mass; and (iv) three-dimensional (3D) structural conformation. EPS are classified as homopolysaccharides (HoPS), consisting of a single type of monosaccharide, or heteropolysaccharides (HePS), composed of two or more types of monosaccharides. HoPS are glucans, fructans, or galactans, made up of repeating units of glucose, fructose, or galactose, respectively (Pérez-Ramos et al., 2015).

The α-D-glucans are the most widely produced HoPS and according to the linkage in the main chain, they are subdivided in dextrans α-(1,6), mutans α-(1,3), reuterans α-(1,4), and alternans α-(1,3) and α-(1,6) and may present different types and degrees of branching (Monsan et al., 2001). Of these, dextrans are currently used in the food and pharmaceutical industries (Aman et al., 2012). The viscosity and rheological properties of the dextran solutions are influenced by their molecular masses and consequently define their applications. Thus, low-molecular mass dextrans are used in the photographic and pharmaceutical industries, whereas the high-molecular mass dextrans are utilized in the chemical industry. Among various medical applications, dextrans are used for anticoagulant therapy as heparin substitutes and blood plasma replacers/expanders. In addition, there is evidence that dextran sulfate has an antiviral effect against human immunodeficiency virus (Piret et al., 2000) and we have recently demonstrated that dextrans synthesized by LAB have potential as antivirals and immunomodulatory agents in trout (Nácher-Vázquez et al., 2015). In the food industry HoPS are added to bakery products and confectionery to improve softness or moisture retention, to prevent crystallization, and to increase viscosity, rheology, texture, and volume (Pérez-Ramos et al., 2015). They are also used as films to protect surfaces of frozen fish, meat, vegetables, or cheese from oxidation and other chemical changes.

Dextrans are synthesized by dextransucrases (Dsr), which catalyze the transfer of D-glucopyranosyl residues from sucrose to the growing polymer, accompanied by fructose release (Werning et al., 2012). The Dsr-encoding genes are carried by strains belonging to the genera *Lactobacillus, Leuconostoc, Oenococcus, Streptococcus, Weissella*, and *Pediococcus* (Kralj et al., 2004a; Naessens et al., 2005; Bounaix et al., 2010; Werning et al., 2012; Amari et al., 2013; Rühmkorf et al., 2013; Dimopoulou et al., 2015; Yanping et al., 2015). Many Dsr have been characterized, but despite the interest of dextrans in various applications, little is known about the regulation of the expression of Dsr. By determining the levels or the activity of the Dsr in bacterial cultures grown in presence of sucrose or other sugars it has been inferred that their expression may be constitutive or sucrose-inducible. However, transcriptional analysis of *dsr* genes expression to validate sucrose inducibility has been performed in a few cases only (e.g., in *Leuconostoc mesenteroides* NRRL B-512F, Quirasco et al., 1999).

We have previously demonstrated that *Lb. sakei* MN1 isolated from a fermented meat product synthesizes an α-(1-6) glucan with ~6% substitution, at positions *O*-3, by side chains composed of a single residue of glucose and with a molecular mass of 1.7 × 10^8^ Da (Nácher-Vázquez et al., 2015; Zarour et al., 2017). We have performed *in vitro* and *in vivo* experiments that support that this dextran has antiviral and immunomodulatory properties of interest in aquaculture (Nácher-Vázquez et al., 2015). Moreover, we have demonstrated that the purified HoPS is able to efficiently immunomodulate *in vitro* human macrophages (Zarour et al., 2017). In addition, we have provided evidences that *Lb. sakei* MN1 has probiotic properties and we have shown that the production of dextran influences, *in vitro*, the bacterial capability for aggregation, biofilm formation, and adhesion to enterocytes as well as *in vivo* bacterial colonization and competition with pathogens in gnotobiotic zebrafish models (Nácher-Vázquez et al., 2017). Therefore this bacterium and its dextran seem to have potential for development of functional synbiotic food and feed. Thus, in this work, we have characterized the genetic basis of dextran production in this bacterium with the aim of having a better knowledge of the practical utility of *Lb. sakei* MN1.

## Materials and methods

### Bacterial strains and growth media

The bacterial strains used in this work are shown in Table 1. The *Latococcus lactis* strains were grown in M17 broth (Oxoid) supplemented with 0.5% glucose (M17G) or 0.5% glucose plus 0.8% sucrose (M17GS) and *Lb. sakei* strains were grown in Man Rogosa Sharpe broth (de Man et al., 1960) with 2% glucose (MRSG) or 2% sucrose (MRSS) or in defined medium with 0.8% glucose (CDMG) or 0.8% sucrose (CDMS) (Sánchez et al., 2008) and incubated at 30°C. *Escherichia coli* strains were grown in LB medium containing 10 g L^−1^ of tryptone, 5 g L^−1^ of yeast extract, and 10 g L^−1^ of NaCl (pH 7.0) and incubated at 37°C. When the bacteria carried pRCR or pRCR-based plasmid derivatives conferring resistance to chloramphenicol (Cm) the medium was supplemented with Cm at 5 μg mL^−1^ for *L. lactis* and *Lb. sakei* strains or at 10 μg mL^−1^ for *E. coli* DH5α.

**Table 1:** Description of bacteria used in this work.

**Strain**	**Plasmids**	**^a^Resistance**	**Information**	**References**
*Lb. sakei* MN1 (CECT 8329)	pMN1 pMN2	–	Isolated from a fermented meat product	Chenoll et al., 2007
*Lb. sakei* MN1[pRCR12]	pMN1 pMN2 pRCR12	Cm^R^	Derivative of pRCR. It contains the promoter Px of *malXCD* operon of *Streptococcus pneumoniae* JNR7/87 fused to the *mrfp* gene	Nácher-Vázquez et al., 2017
*Lb. sakei* MN1[pRCR13]	pMN1 pMN2 pRCR13	Cm^R^	Derivative of pRCR by cloning of region A (GenBank MF590088 position: 38–363) upstream of *mrfp*	This study
*Lb. sakei* MN1[pRCR14]	pMN1 pMN2 pRCR14	Cm^R^	Derivative of pRCR by cloning of region B (GenBank MF590088 position: 1,476–1,819) upstream of *mrfp*	This study
*Lb. sakei* MN1[pRCR15]	pMN1 pMN2 pRCR15	Cm^R^	Derivative of pRCR by cloning of region C (GenBank MF590088 position: 2,392–2,749) upstream of *mrfp*	This study
*L. lactis* MG1363	–	–	Strain derived from *L. lactis* 712 by plasmids curing	Gasson, 1983
*L. lactis* MG1363[pRCR13]	pRCR13	Cm^R^	Derivative of pRCR by cloning of region A upstream of *mrfp*	This study
*L. lactis* MG1363[pRCR14]	pRCR14	Cm^R^	Derivative of pRCR by cloning of region B upstream of *mrfp*	This study
*L. lactis* MG1363[pRCR15]	pRCR15	Cm^R^	Derivative of pRCR by cloning of region C upstream of *mrfp*	This study
*E. coli* DH5α[pRCR]	pRCR	Cm^R^	Promoters probe vector containing the *mrfp* gene, which encodes the fluorescent mCherry protein	Mohedano et al., 2015
*E. coli* V517	Eight plasmids pVA517A through pVA517H	ND	Source of plasmids used as references in agarose gel analysis	Macrina et al., 1978

a *ND, No determined; Cm^R^, resistance to chloramphenicol*.

### Genomic and plasmidic DNA preparations

To isolate plasmidic DNA, bacterial cultures were grown to an absorbance at 600 nm (A_600_) of 2 at 30°C in MRSG and 10 mL of each culture were sedimented by centrifugation (15,700 × g, 10 min, 4°C). For cellular lysis, cells were washed with phosphate buffer saline (PBS, pH 7.4), resuspended in 2 mL of a solution containing 25% sucrose, 30 mg mL^−1^ lysozyme, 120 U mL^−1^ mutanolysin, and 40 μg mL^−1^ RNase A, and incubated for 15 min at 37°C. Then, cell debris and chromosomal DNA were removed from the extracts by: (i) treatment for 7 min at 21°C with 4 mL of a solution containing 0.13 N NaOH and 2% SDS, (ii) incubation for 15 min at 0°C with 3 mL of 1 M potassium acetate pH 4.8, and (iii) sedimentation (centrifugation at 15,700 × g, 15 min, 4°C). The plasmidic DNA present in the supernatants was precipitated and concentrated by addition of 42% (final concentration) isopropanol, centrifugation as above and dissolution in 3.2 mL of ultrapure water. The DNA preparation was purified and deproteinated by treatment with 2 mL of a solution containing 7.5 M ammonium acetate and ethidium bromide at 0.5 mg mL^−1^, addition of 1:1 (v/v) of a mixture of phenol, chloroform, and isoamyl alcohol (50:48:2, vol/vol/vol) and centrifugation (15,700 × g, 10 min, 21°C). Plasmidic DNA was precipitated from the aqueous phase with 69.5% (final concentration) ethanol for 12 h at −20°C and recovered by centrifugation (11,269 × g, 45 min, −10°C). The precipitated DNA was washed with 1 mL of 70% ethanol, sedimented by centrifugation (11,269 × g, 30 min, −10°C), and dissolved in 10 mM Tris buffer pH 8.0 (100 μL).

For the isolation of genomic DNA, bacterial cultures were grown to an A_600_ = 2 at 30°C in MRSG and 1 mL of each culture were sedimented by centrifugation (15,700 × g; 10 min, 4°C). For cellular lysis, cells were washed with phosphate buffer saline (PBS, pH 7.4), resuspended in a solution (100 μL) containing 25% sucrose, 50 mM Tris pH 8.0, 0.1 M NaCl, 30 mg mL^−1^ lysozyme, 240 U mL^−1^ mutanolysin, and 80 μg mL^−1^ RNase A, and incubated for 15 min at 37°C. Then, lysed cells were treated with 1% (final concentration) SDS for 2 min at 21°C and passed through a 25 GA needle (0.5 × 16 mm) three times. The extracts were deproteinated by treatment with an equal volume of a mixture of phenol, chloroform, and isoamyl alcohol (50:48:2, vol/vol/vol) for 5 min at 21°C and centrifugation (15,700 × g, 10 min, 21°C). The DNA contained in the aqueous phase was precipitated with 69.5% (final concentration) ethanol and 83 mM (final concentration) sodium acetate pH 7.0 for 12 h at −20°C and recovered by centrifugation (11,269 × g, 45 min, −10°C). The precipitated DNA was washed with 1 mL of 70% ethanol, sedimented by centrifugation (11,269 × g, 30 min, −10°C), and dissolved in 10 mM Tris buffer pH 8.0 (100 μL).

### Total RNA preparations

For the isolation of total RNA, bacterial cultures were grown to A_600_ = 2 at 30°C in CDMG or CDMS for RT-PCR. Total RNAs were isolated using the kit “FastRNA Pro Blue” (QBIOgene) and subjected to electrophoresis in 0.8% agarose gels at a constant voltage of 135 V for 20 min to check the integrity of the rRNAs. The total RNA concentration was determined with a Qubit 2.0 fluorimeter (Invitrogen) following the instructions of the supplier. To ensure absence of DNA, the RNA preparations were incubated for 1 h at 37°C with 1 μg mL^−1^ DNase I (Sigma-Aldrich) and then purified following the “RNA Cleanup” protocol of the “RNeasy Midi” kit from QIAGEN. In addition, the samples were subjected to three cycles of deproteinization, which involved incubation with acid phenol for 5 min at 70°C with shaking and subsequent centrifugation (15,700 × g, 21°C, 5 min). The collected supernatants were treated with one volume of phenol:chloroform-isoamyl alcohol (50:48:2) and centrifuged at 15,700 × g, 21°C, 10 min). The RNA present in the aqueous phase was precipitated by the addition of 1/10 volume of 3 M sodium acetate (pH 7.0) and three volumes of absolute ethanol, followed by storage at −20°C for 12 h. The precipitated RNA was recovered by centrifugation (11,269 × g, −10°C, 45 min), washed with 1 mL of 75% ethanol and finally dissolved in 200 μL of diethylpyrocarbonate-treated water.

### Detection of the genes

#### Oligonucleotides, PCR, RT-PCR, and sequencing

The oligonucleotides used are summarized in Table 2. The nucleotide sequences of 14 bacterial genes encoding dextransucrase enzymes, obtained from GenBank of the National Centre for Biotechnology (NCBI, USA), were analyzed with the BLAST program in order to design the primers dsrF and dsrR for further amplification of a conserved dextransucrase coding region, located at the catalytic site of these enzymes. Genomic DNA and plasmidic DNA of *Lb. sakei* MN1 were used as templates in a reaction with primers dsrF and dsrR with Phusion Hot Start High Fidelity Polymerase (HSHFP, ThermoFisher Scientific) following the instructions of the enzyme supplier.

**Table 2:** Description of primers used in this work.

**Target**	**Primer^a^**	**Sequence (5′-3′)**	**Product (bp)**
*pcrA*	pcrF	ACAAACATGGCGCATCAACG	146
	pcrR	GCGAAGGTGCTCAAGATGTTT	
*repA*	repF	GGCAAGCCGGTTATTGGTTAC	140
	repR	TTTTCCTGCTCTGTTAATTCACCAT	
*dsrLS*	dsrF	GATGATGGTCAATATATGGCAA	695
	dsrR	CTTGAACGATATTGTGGTGCCAA	
Region A	P1F	GAAGATCTTCTTTTAGACCCCTCTTGAGGCT	343
	P1R	GCTCTAGAGCAGTATCATCACCTTTATCGCGC	
Region B	P2F	GGAAGATCTTCCTCAGCAACAACGGTTAGCCT	366
	P2R	GCTCTAGAGCCGCCAGTGATCATATAACCGA	
Region C	P3F	GGAAGATCTTCCAAATTAACCAGAGACCGC	376
	P3R	GCTCTAGAGCTGGCTGGCTGGTAACTAGCA	
RT-PCR Amplicon 1	rt1F	AGCTGGGTTCGATATGCTTTA	1,150
	rt1R	CCCACCCCTCGCTCTTTA	
RT-PCR Amplicon 2	P2F	GGAAGATCTTCCTCAGCAACAACGGTTAGCCT	1,952
	rt2R	CGGTTGGCAAAGACGTTTTG	
RT-PCR Amplicon 3	P3F	GGAAGATCTTCCAAATTAACCAGAGACCGC	1,033
	rt2R	CGGTTGGCAAAGACGTTTTG	
RT-PCR Amplicon 4	rt2F	ACGGCTGCGATCACTACTG	496
	rt2R	CGGTTGGCAAAGACGTTTTG	
RT-PCR Amplicon 5	rt3F	AGCTTACGCTGCTACCAAGGC	862
	rt3R	ATGGCTGGAGTAAAATGGATCAGCT	

a *F, Forward; R, reverse*.

For RT-PCR, total RNA preparations were used. For the synthesis of the cDNAs, 400 ng of RNA, 0.75 μM of each of the oligonucleotides complementary to different regions of pMN1 and 500 μM of each of the 4 dNTPs (dATP, dGTP, dCTP, and dTTP) were used. The mixtures were incubated for 5 min at 65°C, transferred to 4°C and to each were added 1X cDNA synthesis buffer (Invitrogen), 5 mM DTT (Invitrogen), 2 U μL^−1^ of RNaseOUT (Invitrogen), and 0.75 U μL^−1^ of avian reverse transcriptase from ThermoScript (Invitrogen). The reaction was allowed to proceed for 1 h at 50°C and samples were next incubated 5 min at 85°C to stop the reaction. To remove RNA residues, 0.2 μg μL^−1^ of RNase A (Sigma-Aldrich) was added and the mixture was incubated for 20 min at 37°C. Finally, the samples were dialyzed for 30 min against 10 mM Tris pH 8.0 using membranes of the Millipore V series (Merck). PCRs were carried out according to the instructions of the Thermo Fisher Scientific Phusion DNA polymerase using the primers described in Table 2.

DNA sequencing was performed by the dideoxynucleotide method at Secugen (Madrid, Spain). For the determination of the nucleotide sequence of the pMN1 plasmid, the walking strategy was followed, after detection of the *dsrLS* gene by PCR with the dsrF and dsrR primers and further sequencing of the amplicon. To determine the sequence of pMN1 by the dideoxynucleotide method, it was used as substrate the product of a polymerization reaction catalyzed by the bacteriophage Φ29 DNA polymerase with plasmidic DNA of *Lb. sakei* MN1 and hexamers containing random sequences. The DNA sequence of plasmid pMN1 was deposited in GenBank (accession No MF590088).

#### Southern blot hybridization

Genomic and plasmidic DNA preparations were subjected to electrophoresis in a 0.7% agarose gel with 40 mM Tris, 20 mM acetate, and 1 mM EDTA buffer at constant amperage of 40 mA for ~4 h, and DNA molecules were revealed by staining with ethidium bromide at 0.5 μg mL^−1^. Then, DNA was transferred to a 0.45 μm nylon membrane (Biodyne A, Pall Corporation) and hybridized with the probe. The temperature of hybridization was 45°C. Probe labeling and detection procedures were performed with the NEBlot® Phototope® Kit (New England BioLabs) and the Phototope®-Stars Detection Kit (New England Biolabs). Substrate for probe labeling was the 695-bp amplicon synthesized with primers dsrF and dsrR (Table 2) and *Lb. sakei* MN1 genomic DNA following the indications of the kit supplier.

### Determination of pMN1 plasmid copy number

#### Preparation of template DNA for real time-qPCR

Exponential cultures (A_600_ = 1.0) of *Lb. sakei* MN1 grown in either MRSG or MRSS at 30°C were generated by inoculation of the media (dilution 1/1,000) with a bacterial stock culture previously grown in MRSG. These two cultures (designated generation 0) were always maintained in the exponential phase and sub-cultured in the corresponding medium by dilutions 1/1,000 for 60 more generations. Then genomic DNA was isolated from ~0.5 × 10^9^ bacteria of the 0 and 60 generations cultures by using the Wizard® Genomic DNA Purification Kit (Promega). At the cell lysis step, 30 mg mL^−1^ of lysozyme and 30 U of mutanolysin were added. Concentration of the genomic DNA was determined with a Qubit fluorometer by using the Qubit HS dsDNA Assay Kit (Molecular Probes). The DNA extracts were digested with EcoRI, a restriction enzyme that linearizes the pMN1 plasmid leaving intact the *repA* and *pcrA* amplicons. This method was developed to obtain accurate qPCR-based plasmid copy numbers (Providenti et al., 2006).

#### Real time-qPCR analysis

Two primer sets were designed, based on the pMN1 sequence (this work) and on the *Lb. sakei* 23K (NC_007576.1) *pcrA* sequence, specific for either the pMN1 replication protein coding gene (*repA*) or the PcrA helicase chromosomal reference gene (*pcrA*). The criteria used during primer design was that the primers had a predicted Tm of ~59°C and that they generated amplicons ~140-bp long.

The qPCR were conducted in a total volume of 20 μL using an iQ5 real-time detection system (BIO-RAD) and the IQ™ SYBR® Green Supermix (Bio-Rad Laboratories), following the manufacturer's recommendations. Decimally diluted EcoRI-digested total DNA preparations (15, 1.5, 0.15, and 0.015 ng per reaction) were analyzed using 0.5 μM (final concentration) of the specific forward and reverse primers. To prepare the reactions and minimize pipetting errors 2 μl of template DNA were added to individual qPCRs. Thermal cycling conditions were as follows: initial denaturation at 95°C for 5 min, followed by 40 cycles of 95°C for 10 s (denaturation), 59°C for 30 s (primer annealing), and 72°C for 20 s (elongation). A melting curve analysis of the PCR products, with a temperature gradient of 0.1°C/s from 59 to 95°C, was performed to confirm the purity and specificity of the PCR products. Two independent qPCR trials were conducted for each template source. In each trial, triplicate samples of the four different amounts of template were analyzed.

Relative copy number of pMN1 was calculated using equation (1):

(1) $$PCN={\left(1+\;E_{pcrA}\right)}^{Ct_{pcrA}}/{\left(1+\;E_{repA}\right)}^{Ct_{repA}},$$

where *E*_*pcrA*_ and *E*_*repA*_ are, respectively, the PCR amplification efficiencies of the chromosomal and plasmidic amplicons, and *Ct*_*pcrA*_ and *Ct*_*repA*_ are the mean threshold cycle values obtained for the corresponding amplicons. A PCN value was calculated for each of the four template concentrations analyzed, and the average and standard deviation of the four values was estimated.

E values of target (*E*_*repA*_) and reference (*E*_*pcrA*_) sequences were empirically calculated for each qPCR trial. For that purpose, mean Ct values were plotted against the logarithm of the amount of total DNA template in the assay (**Figure 2C**). From the slope of the curve generated by linear regression of the plotted points, the PCR amplification efficiency was determined according to the equation:

(2) $$E=\;10^{-1/slope}-1$$

Although the *E* values for both amplicons was higher than 0.9, we have chosen Equation (1) to calculate the relative plasmid copy number as it allows taking into account the slight differences between E_target_ and E_reference_ that we have observed.

### Construction of pRCR-pMN1 derivative plasmids to detect promoter regions driving transcription of *dsrLS*

The pRCR vector containing the *mrfp* gene, which encodes the fluorescent mCherry protein, was used to detect and evaluate the performance of pMN1 promoter(s) of *dsrLS*. For the construction of pRCR13, pRCR14, and pRCR15 (pRCR derivatives, Figure S1), amplicons of 343-, 366-, and 376-bp were synthesized by PCR with HSHFP following the instructions of the DNA polymerase supplier. The substrate for the reaction was pMN1 present in a plasmidic DNA preparation of *Lb. sakei* MN1 and the following oligonucleotide pairs, P1F and P1R for pRCR13; P2F and P2R for pRCR14; P3F and P3R for pRCR15 (Table 2). The plasmid vector pRCR, obtained from *E. coli* DH5α[pRCR], and the amplicons generated by PCR were subjected to digestion with BglII and XbaI (New England Biolabs) and ligated into the pRCR vector with the T4 DNA ligase (New England Biolabs) to obtain the recombinant plasmids. The ligations mixtures were used to transform *L. lactis* MG1363 by electroporation (25 μF, 2.5 kV and 200 Ω in 0.2 cm cuvettes), as previously described (Dornan and Collins, 1987), and transformants were selected in M17G agar plates supplemented with Cm at 5 μg mL^−1^. The three new plasmid constructs were confirmed by automated sequencing. DNA preparations of pRCR13, pRCR14, and pRCR15 obtained from *L. lactis* MG1363 were then used to transform *Lb. sakei* MN1 by electroporation (25 μF, 1.8 kV and 600 Ω in 0.2 cm cuvettes) as previously described (Berthier et al., 1996) and transformants were selected in MRSG-agar plates supplemented with Cm at 5 μg mL^−1^.

### Detection of mCherry fluorescence in LAB carrying pRCR12, pRCR13, pRCR14, or pRCR15

To detect the expression of the mCherry fluorescent protein, *L. lactis* strains containing pRCR12, pRCR13, pRCR14, or pRCR15 were grown in 10 mL of M17G or M17GS till the initial stationary phase (A_660_ = 2.5–2.6) or to late stationary phase (A_660_ = 3.0–3.2). *Lb. sakei* strains containing the same plasmid constructs were grown in 10 mL of MRSG or MRSS medium to middle exponential phase (A_600_ = 2) or to late exponential phase (A_600_ = 5 or A_600_ = 10 for cultures grown in MRSG or in MRSS, respectively). All cultures were centrifuged (16,000 × g, 15 min, 4°C), resuspended in PBS buffer (pH 7.4), sedimented by centrifugation as above and finally resuspended in 500 μL of PBS buffer (pH 7.4). Suspensions (200 μL of each) were used to measure the fluorescence levels of the mCherry protein in a Varioskan Flash (Thermo Fisher Scientific) equipment, using 587 and 610 nm wavelengths for excitation and emission detection, respectively. In addition, appropriate dilutions were prepared to estimate culture biomass by measuring the absorbance at A_600_ or A_480_ for *L. lactis* or *Lb. sakei*, respectively. Three independent trials were performed and the same fresh suspensions (8 μL of each), without fixing, were used for phase contrast and fluorescent microscopy analysis as previously described (Nácher-Vázquez et al., 2017). A Leica AF6000LX-DMI6000B model microscope (Leica Microsystems, Mannheim, Germany) was used. Illumination was provided with a 100 × objective. For detection of mCherry BP 620/60 excitation and BP 700/75 emission filters were used. Image analysis was performed using LAS AF CoreSoftware (Leica Microsystems).

### Bioinformatic analysis of DNA sequences and modeling of Dsr

The DNA sequence of plasmid pMN1 was analyzed with the programs included in the DNASTAR Lasergene 12 (DNAstar Inc.). Homologies of pMN1 DNA sequences and of its inferred translated products with the NCBI data bases of the National Center for Biotechnology Information (NCBI) were analyzed with the Basic Local Alignment Search Tool (BLAST) (https://blast.ncbi.nlm.nih.gov/Blast.cgi). Multiple sequence alignment of genes and proteins were performed with the Megaling (DNASTAR laser gene 12) and Clustalx 2.1 (http://www.ebi.ac.uk/Tools/msa/clustalw2/) programs.

The primers for the qPCR experiments were designed with Primer3 v0.4.0 (Koressaar and Remm, 2007; Untergasser et al., 2012).

The modeling of the DsrLS was generated based on its homology with the glucansucrase of *Lactobacillus reuteri* 180, using the 3D-structure of the GTF180-ΔN glucansucrase and the I-TASSER program (Roy et al., 2010). The superposition of the DsrLS model and the 3D-structure of GTF180-ΔN was performed with the CE program at the http://source.rcsb.org.

## Results

### Detection and genomic localization of *dsrLs*

Previous characterization of the EPS produced by *Lb. sakei* MN1 revealed that it is a dextran (Nácher-Vázquez et al., 2015) and indicated that this bacterium produces a Dsr (named DsrLS) responsible for the polymer synthesis. Thus, based on the known sequences of *dsr* genes from other bacteria, primers dsrF and dsrR were designed to amplify by PCR a DNA fragment located at the coding sequence of the dextransucrase catalytic domain. The expected amplicon (695-bp) was obtained using either genomic or plasmidic DNA preparations from *Lb. sakei* MN1 as template (data not shown). The determination of the nucleotide sequence of this amplicon (GenBank accession No KJ161305) and its BLAST analysis against the nucleotide (nr/nt) data base of NCBI revealed only high homologies (100, 99, and 71% identity, with 40 gaps) with genes encoding dextransucrases of *Lactobacillus curvatus* TMW1624 (GenBank accession No HE972512), *Lb. sakei* Kg15 (GenBank accession No AY697434) and *Weisella confusa* strain Cab3 (GenBank accession No KP729387.1), respectively. The overall results supported that the *dsrLS* gene has been detected, and, generation of the expected amplicon using the plasmidic DNA preparation strongly suggested that it was plasmid-borne. In addition, they revealed that the dsrF and dsrR primers pair designed by us are useful for the detection of *dsr* genes in a similar way to those degenerate oligonucleotides previously used for the detection of genes encoding glucosyltransferases of *Lactobacilli* (Kralj et al., 2003).

**Figure 1:**
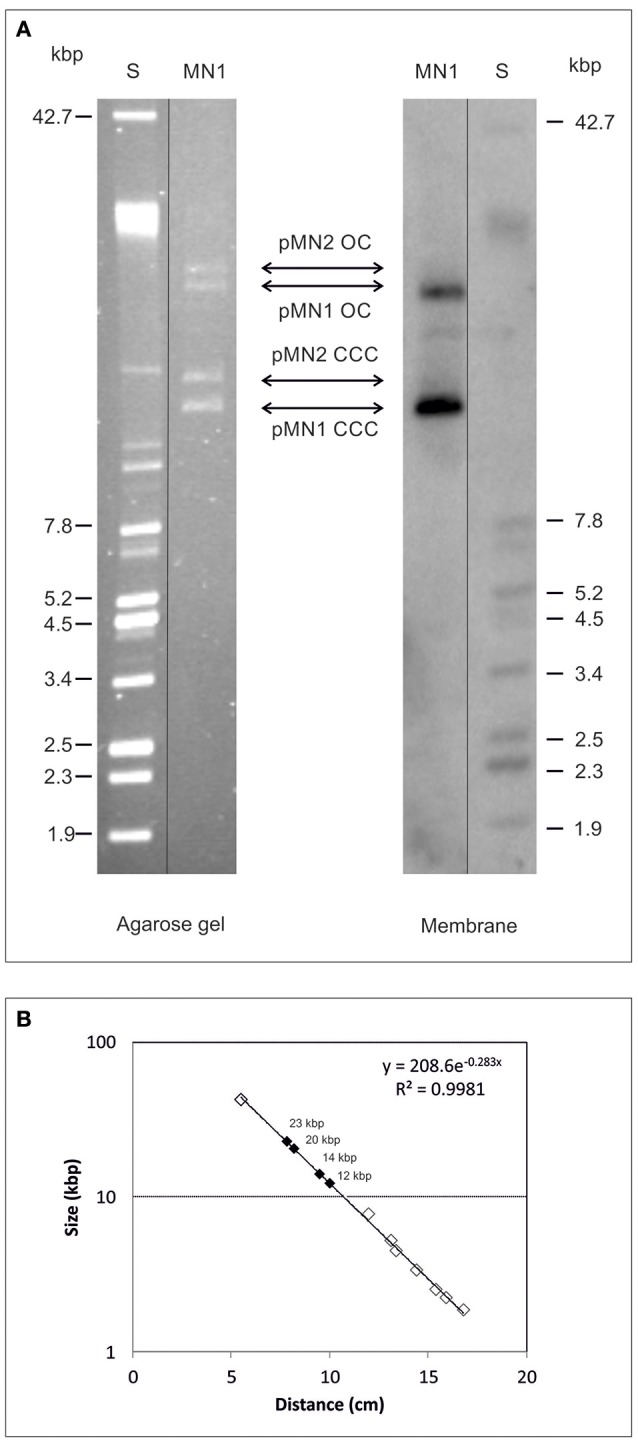
Detection of *dsrLS* gene by Southern blot hybridization. Plasmids preparations of *Lb. sakei* MN1 (MN1) and of *E. coli* V517 (S) were analyzed in 0.7% agarose gel (**A** left) transferred to a membrane and hybridized for detection of *dsrLS* (**A** right). In **(B)**, the calibration curve for plasmid size determination is depicted.

Analysis of a plasmidic DNA preparation from *Lb. sakei* MN1 in agarose gel revealed the presence of two groups of two bands each (Figure 1A). In addition, it was detected that exposure of the DNA preparations to repeating freezing (at −80°C) and thawing (at 4°C) cycles resulted in alteration of the intensity of the bands: an increase in the two upper bands accompanied by a decrease in the two faster migrating ones (results not shown). The sizes of the bands were inferred from their migration using a calibration curve (Figure 1B) generated with the plasmids of the *E. coli* V517 strain (Figure 1A, lane S), and the overall results indicated that bands with higher mobility could be the covalently closed circles of two plasmids of ~12 and 14 kbp named respectively pMN1 and pMN2, whereas bands with less mobility could be the open circle forms of those plasmids. To disclose the location of *dsrLS*, the 695-bp amplicon was used as a probe for Southern blot hybridization and the blot revealed the presence of two hybridization bands corresponding to the two proposed pMN1 forms (Figure 1A).

### Characterization of plasmid pMN1

**Figure 2:**
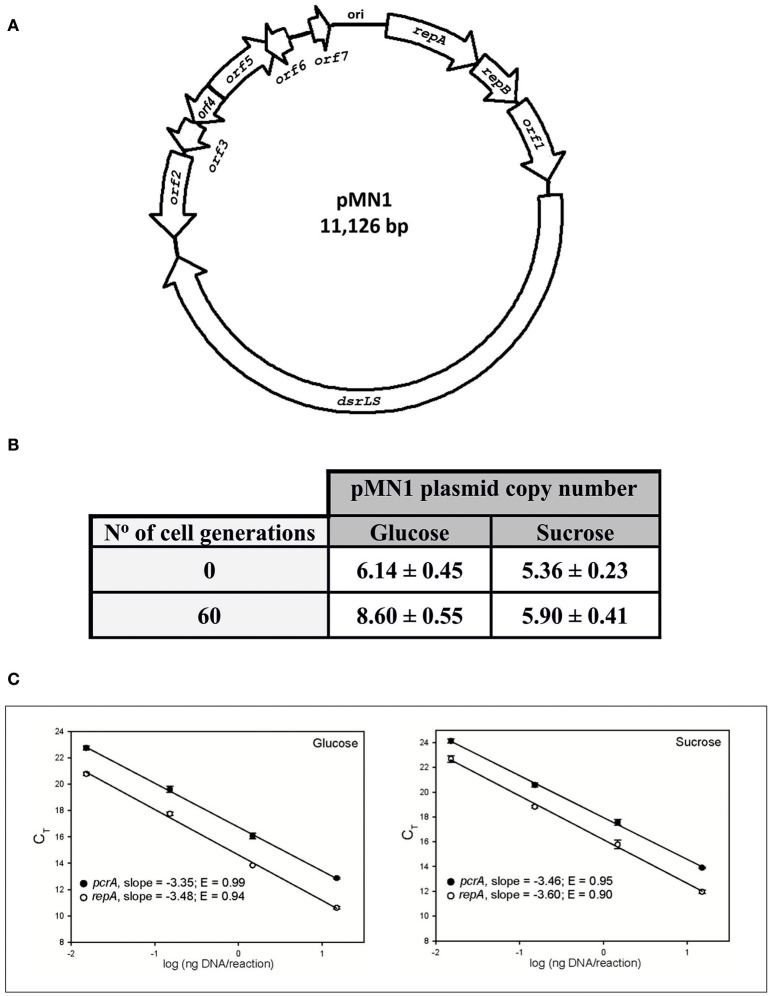
Genetical map of plasmid pMN1 **(A)** and determination of its copy number **(B,C)**. **(A)** The genes and *orf* of pMN1 are depicted. **(B)** Results of the pMN1 copy-number determination are summarized in this table. **(C)** For each gene, determined mean Ct values were plotted against the logarithm of the four amounts of template DNA used in the qPCR assays. PCR amplification efficiencies (E values) were calculated from the slope of the curves generated by linear regression through the experimental points.

Following DNA sequencing, the size of pMN1 plasmid was estimated as 11,126 kbp. A genetic map of pMN1 is depicted in Figure 2A. Blast alignment of its DNA sequence with those deposited in Genbank lead to confirm the existence of the 5,304-bp *dsrLS* gene. Moreover, in pMN1, a 1,674-bp replicon homologous to the replicons of the pUCL287 plasmid family was identified that replicates bidirectionally by theta mechanism (Benachour et al., 1995). This region contains the origin of replication and the *repA* and *repB* genes. In addition, in pMN1, seven other open reading frames were identified and designated ORFs 1-7, which could encode hypothetical proteins. Blast analysis of inferred amino acid sequence of the ORFs with those deposited in the Non-redundant protein sequences database reveled that *orf1, orf3, orf4*, and *orf5* could encode respectively a truncated type I restriction endonuclease subunit R, a transcriptional regulator belonging to the XRE family, a RelE type toxin addiction module and a site-specific integrase.

Furthermore, the copy number of the plasmid was investigated by real time-qPCR by analysis of the *repA* gene vs. that of the chromosomal housekeeping and monocopy *pcrA* gene. *Lb. sakei* MN1 cultures were maintained in exponential growth phase in either MRSG or MRSS and DNA preparations of cultures grown to exponential phase once (from the glycerol stock) or for 60 successive generations (by six subsequent 1/1,000 dilutions) were analyzed. The results are summarized in Figure 2B, and revealed that pMN1 is a low-copy-number plasmid (~6.5 ± 1.5 copies per genome equivalent), which maintains its copy number over at least 60 generations, and that its copy number is not significantly affected by conditions required for dextran synthesis.

### Gene expression of *dsrLs*

**Figure 3:**
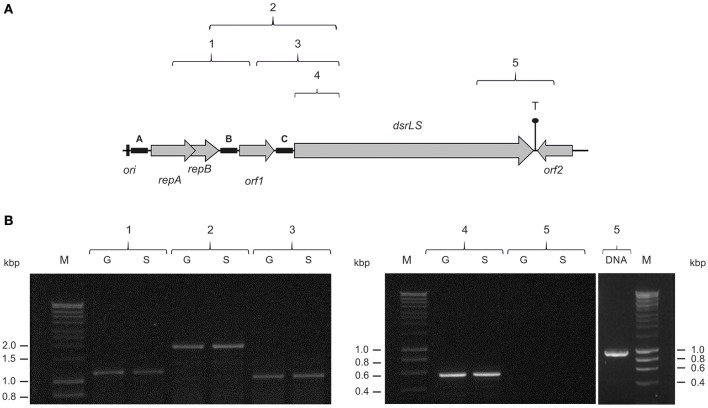
Detection of *dsrLS* transcription by RT-PCR. **(A)** The genes and *orfs* of pMN1 are depicted. The locations of the five amplicons (1, 2, 3, 4, and 5) in pMN1 are shown. **(B)** Analysis in 0.7% agarose gel of the RT-PCR reactions performed using total RNA obtained from cultures of *Lb. sakei* MN1 grown in presence of glucose (G) or sucrose (S) and PCR reaction performed with plasmidic DNA to generate amplicon 5 (DNA). M, DNA standard marker (SmartLadder, Eurogentec).

We have previously detected that *Lb. sakei* MN1 is unable to synthesize the dextran in the absence of sucrose (Nácher-Vázquez et al., 2017). This could be due not only to the lack of the substrate for the polymer synthesis, but also to the fact that *dsrLS* gene expression requires an induction mediated by the disaccharide as it has been detected in other LAB (Neubauer et al., 2003). In addition, inspection of the pMN1 DNA sequence indicated that transcription of *dsrLS* could be driven from more than one promoter located upstream of this gene. Thus, total RNA preparations were obtained from *Lb. sakei* MN1 cultures grown in medium containing either glucose or sucrose and five RT-PCR reactions were performed to generate the amplicons showed in Figure 3A. These amplicons contain regions of more than one gene and their corresponding intergenic regions (amplicons 1, 2, 3, and 5) or only a region of *drLS* (amplicon 4). The results revealed that four reactions generated the expected sizes 1, 2, 3, and 4 amplicons, which included regions located upstream of, or within, the *dsrLS* gene (Figure 3B). In addition, the quantification of the intensity of the amplicons (results not shown) indicated that the mRNA levels were very similar in cultures grown in CDMG or CDMS media. The fifth reaction did not reveal the amplicon 5 (Figure 3B), which carries the 3′-end of *dsrLS* and downstream regions (Figure 3A). This latter result was not due to the primers used, since the expected amplicon was obtained using the plasmidic DNA preparation as substrate (Figure 3B). In addition, the negative result was expected due to the convergent polarity of *dsrLS* and *orf2* and to the existence of a putative bidirectional transcriptional terminator located between the 3′-ends of the two genes, which predicted the lack of their co-transcription.

### Identification of promoter regions in pMN1

As expected, the RT-PCR analysis revealed that *repA* and *repB* were co-transcribed, but in addition showed the existence of transcripts including *dsrLS* and upstream genes. Therefore, detection of DNA regions involved in *dsrLS* expression was approached. We have previously developed the pRCR promoter probe plasmid (Figure S1) based on the pSH71 replicon, which replicates *via* a rolling circle mechanism and carries a mCherry-coding gene (*mrfp*) optimized for expression of the fluorescent protein in LAB. Moreover, we had shown functionality of this replicon concomitant with successful expression of this *mrfp* in *L. lactis* (Mohedano et al., 2015) and *Lb. sakei* MN1 (Nácher-Vázquez et al., 2017). Thus, to detect promoter regions that could drive transcription of *dsrLS* in *L. lactis* and *Lb. sakei*, A, B, and C DNA fragments, carrying intergenic regions located upstream of the *repA, orf1*, and *dsrLS* genes, respectively (Figure 3), were cloned upstream of the *mrfp* gene into the pRCR plasmid, generating the recombinant plasmids pRCR13, pRCR14, and pRCR15. Thus, these plasmids carry putative transcriptional fusions to the mCherry-coding gene (Figure S1).

The clonings were performed in *L. lactis* MG1363 and then the plasmids were transferred to *Lb. sakei* MN1. Afterwards, expression of the mCherry in the two hosts was monitored by fluorescent spectroscopy and microscopy. *L. lactis* cannot grow in media containing sucrose as the only carbon source. Therefore, expression of mCherry was monitored during growth in M17G and M17GS. The results obtained during the early and late stationary phases are shown in Table 3 and Figure S2. As expected, MG1363 did not show fluorescence. Moreover, fluorescence was observed only in cultures of MG1363[pRCR13] and MG1363[pRCR15] and the promoter regions present in them were designated P1 and P2, respectively. Nevertheless, the levels of fluorescence in these strains were low and only significantly detected at late stationary phase. This pattern of expression had been previously observed when the *mrfp* gene was expressed in *L. lactis* MG1363 under control of a lactococcal promoter (Garcia-Cayuela et al., 2012), and it could indicate that the mCherry protein requires a long period of maturation prior to emit fluorescence in this host.

**Table 3:** Fluorescent detection of promoter regions in *L. lactis* by translational fusions to the *mrfp* gene.

**Growth phase**	**^a^Initial stationary phase**		**^a^Late stationary phase**	
** 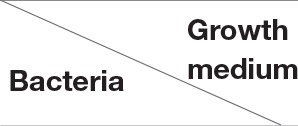 **	**M17G**	**M17GS**	**M17G**	**M17GS**
*L. lactis* MG1363	0.0 ± 0.0	0.0 ± 0.0	0.2 ± 0.1	0.10 ± 0.0
*L. lactis* MG1363 [pRCR13]	0.7 ± 0.5	0.8 ± 0.1	16.6 ± 0.8	12.5 ± 1.2
*L. lactis* MG1363 [pRCR14]	0.2 ± 0.1	0.1 ± 0.0	0.2 ± 0.0	0.1 ± 0.0
*L. lactis* MG1363 [pRCR15]	0.6 ± 0.2	0.9 ± 0.1	10.2 ± 0.8	10.4 ± 1.6

a *The specific fluorescence is depicted and it was calculated as the ratio of the detected fluorescence (10 ×) and the bacterial biomass estimated from the A_600_ of the culture*.

**Figure 4:**
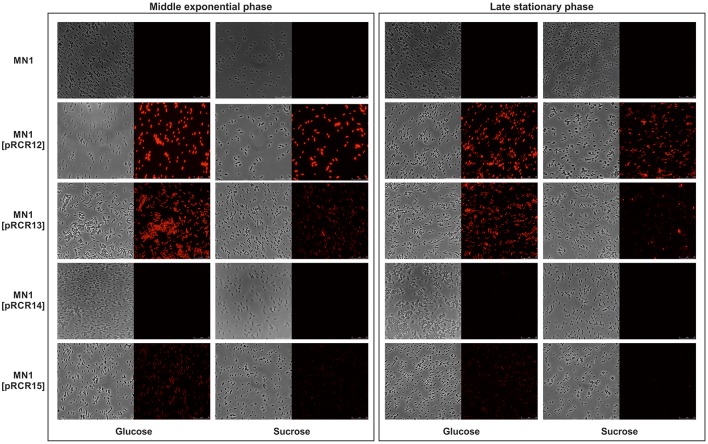
Detection of fluorescence in *Lb. sakei* strains. Cultures of the indicated strains in MRSG (glucose) or MRSS (sucrose) were analyzed at middle exponential and late stationary phases by phase contrast **(Left)** or fluorescence **(Right)** microscopy.

In the case of *Lb. sakei*, strains were grown in MRSG and MRSS and the presence of pRCR13 and pRCR15, and not of pCRC14, conferred fluorescence to *Lb. sakei* MN1, in both exponential and stationary phases (Table 4 and Figure 4). The levels of specific florescence indicated that P1 is stronger than P2 and that they are weaker than the pneumococcal Px promoter, which drives transcription of *mrfp* in *Lb. sakei* MN1[pRCR12] (Table 4) (Nácher-Vázquez et al., 2017).

**Table 4:** Fluorescent detection of promoter regions in *Lb. sakei* by expression of translational fusions to the *mrfp* gene.

**Growth phase**	**^a^Middle exponential phase**		**^a^Late stationary phase**	
** 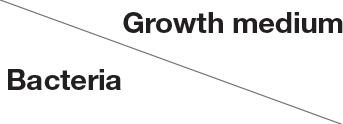 **	**Glucose**	**Sucrose**	**Glucose**	**Sucrose**
*Lb. sakei* MN1	1.3 ± 0.6	0.9 ± 0.1	0.5 ± 0.1	0.4 ± 0.5
*Lb. sakei* MN1[pRCR12]	355 ± 30	351 ± 18	227 ± 14	139 ± 5.5
*Lb. sakei* MN1[pRCR13]	142 ± 14	76 ± 7.5	149 ± 11	68 ± 2.2
*Lb. sakei* MN1[pRCR14]	4.6 ± 0.4	2.0 ± 0.2	5.5 ± 0.3	1.6 ± 0.1
*Lb. sakei* MN1[pRCR15]	73 ± 2.6	28 ± 2.0	40 ± 0.9	8.7 ± 0.4

a *The specific fluorescence is depicted and it was calculated as the ratio of the detected fluorescence (10 ×) and the bacterial biomass estimated from the A_600_ of the culture*.

In addition, at the exponential phase, the specific fluorescence in MN1[pRCR13] and MN1[pRCR15] strains was two-fold-higher in MRSG that in MRSS. These results confirmed that the sucrose present in the medium is not an inducer of the *dsrLS* gene.

## Discussion

The enzymes responsible for the HoPS synthesis are glycosyl hydrolases, extracellular polymerases that utilize the energy of the glucosidic bond of sucrose to link molecules of glucose. If they synthesize α-D-glucans are called glucansucrases and according to the CAZy classification (http://www.cazy.org), are members of the GH70 family. Among them, dextransucrases synthesize dextran and here we have characterized the *Lb. sakei* MN1 *dsrLS* gene of 5,304-bp, which encodes the DsrLS composed of 1,767 amino acids (aa). Analysis of these sequences with the BLAST program vs. those deposited in the NCBI databases revealed homologies with other bacterial genes and with their gene products. The highest homology was detected with the 5,094-bp *gtf1624* gene from *Lb. curvatus* TMW1624 and its product, the dextransucrase GTF1624 of 1,697 aa (Rühmkorf et al., 2013). In addition, the *dsrLS* gene and DsrLS also exhibited high homology with the *gtfkg15* gene of *Lb. sakei* Kg15 of 4,788-bp and its product GTFKg15 of 1,595 aa (Kralj et al., 2004a). An alignment of the three proteins is presented in Figure S3.

It has been demonstrated that the EPS synthesized by GTF1624 (Rühmkorf et al., 2013) and GTFKg15 (Kralj et al., 2004a) are α-(1-6)-glucans with a low percentage of substitutions at positions *O*-3, like the EPS synthesized by *Lb. sakei* MN1 (Nácher-Vázquez et al., 2015). This fact strongly supports that indeed DsrLS is the enzyme responsible for the synthesis of the MN1 dextran.

In all glucansucrases, including dextransucrases, exist: (i) a N-terminal, variable region, (ii) the catalytic domain, and (iii) a C-terminal, so-called “glucan binding” domain (van Hijum et al., 2006). All these regions were identified in DsrLS and include the following aa: (i) 49–390, (ii) 391–1,154, and (iii) 1,155–1,767. The glucansucrases are extracellular enzymes, and at the N-terminus of DsrLS it was found a sequence characteristic of the leader peptides of Gram-positive bacteria (1–48 aa), also present in GTF1624 and GTFKg15. In addition, the difference in the number of aa of the three dextransucrases is due in one hand to the absence in GTF1624 of 70 aa present in the C-terminal region of DsrLS (residues 1,541–1,610). On the other hand, DsrLS has 172 aa more than its homolog of *Lb. sakei* Kg15, being 145 of them located at the C-terminal region (1,485–1,629) and 27 aa at its N-terminal variable region (residues 64–89 and 813). Consequently, the greatest divergence of DsrLS with both GTF1624 and GTFKg15 is located at its C-terminal domain.

Currently, the 3D-structure of any entire glucansucrase has not been determined, but partial structures of: (i) the DSR-E-ΔN of *Lc. mesenteroides* NRRLB-1299 (PDB 3TTQ), (ii) the GTF-S1 of *Streptococcus mutants* (PDB 3AIE), (iii) the GTFA-ΔN of *Lb. reuteri* 121 (PDB 4AMC), and (iv) the GTF180-ΔN of *Lb. reuteri* 180 (PDB 3KLK, 4AYG, and 3HZ3) have been solved by X-ray diffraction analysis of crystals. From the crystal structure of these proteins, it has been stablished that there are five structural domains designated A, B, C, IV, and V (Leemhuis et al., 2013). The A, B, and C domains have been named following the nomenclature of the structurally homologous domains of the GH13 family of α-amilases. The domains IV and V have not homologs in GH13 and for this reason have been named with a different nomenclature (Vujicic-Zagar et al., 2010). These domains are not adjacent in the primary structure of the proteins, and are located with a “U” distribution and a pattern V, IV, B, A, C, A, B, IV, and V.

**Figure 5:**
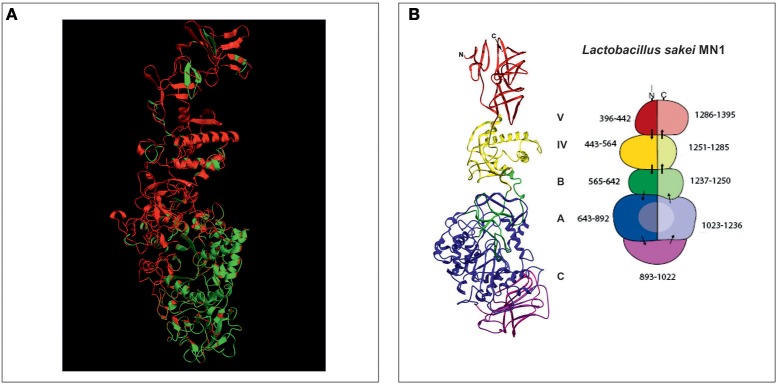
3D-model of the structure of DsrLS. **(A)** Superposition of the structural model of DsrLS (in red) on the crystal structure of GTF180-ΔN (in green). **(B)** The five structural domains of DsrLS as well as the numbering of the corresponding aa are shown. The color codes are: blue for A, green for B, violet for C, yellow for IV, and red for V.

**Figure 6:**
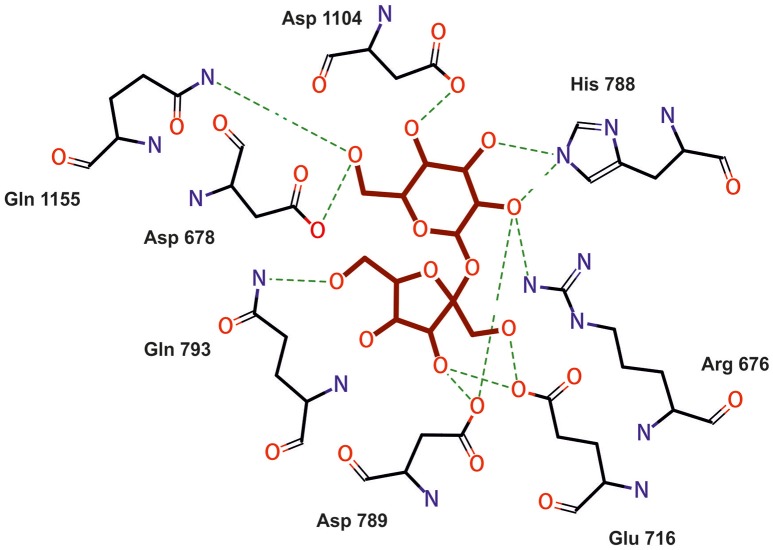
Model of amino acids interaction with sucrose (magenta) at the catalytic center of DsrL. Model obtained by superimposing the model structure of DsrLS on the co-crystal of GTF180-ΔN and sucrose.

The aa sequence of DsrLS (residues 396–1,395) has an identity of 51% with that of the GTF180-ΔN of *Lb. reuteri* 180 (PDB 3HZ3) (Figure S4). Thus, it was possible to develop a model of the 3D-structure of DsrLS lacking the N- and the C-terminal regions (Figure 5), which predicts that the *Lb. sakei* enzyme has the same domains as its homonymous of *Lb. reuteri* (Figure 5B). The A domain is a barrel (β/α)_8_ and contains the catalytic site of the enzyme, including an amino acid triad composed of two aspartate and one glutamate residues, which are involved in the formation of a covalent glucosyl-enzyme intermediate, the key step in the transfer of D-glucosyl units. From this intermediate, the glucosyl unit is transferred to the acceptor (the growing dextran molecule) by a processive catalytic mechanism. Thus, superposition of the 3D-model of DsrLS on the co-crystal of GTF180-ΔN and sucrose indicates that D678, D789, and E716 constitute the catalytic triad of the *Lb. sakei* MN1 dextransucrase (Figure 6).

It has been shown that calcium is essential for GTFA-ΔN (Kralj et al., 2004b) and GTF180-ΔN (Vujicic-Zagar et al., 2010) activities. The B domain located adjacent to the A domain seems to be essential for glucansucrases activity because (i) calcium binding site includes aa from A and B domains, and (ii) some elements of the B domain contribute to stabilize the conformation of the A domain. Concerning to the C domain, although is conserved in all glucansucrases, its function is still unknown. The IV domain connects the B and V domains and seems to act as a hinge to bring the V domain close to the catalytic domain (Ito et al., 2011). The V domain is constituted by the N- and C-terminal regions, which include a series of structural modules with two or three β2/β3 units containing ~20 aa and arranged in a regularly repeating fashion, resulting in a β-solenoid fold. In some glucansucrases these modules include YG repeats (containing a tyrosine/glycine motif) (Leemhuis et al., 2013) and DsrLS carries YG in both terminal regions.

In *Lactobacillus*, the variable N-terminal region of glucansucrases contains 200–700 aa and mutations or deletions of this region can alter the functions of the proteins. Thus, in GTFA, deletion of this region affects the interplay between the hydrolytic and transglycosidase activities (Kralj et al., 2004b). The C-terminal domain contains ~300 aa and the function of this region is still unknown. In GTFA, its deletion diminishes affinity for sucrose (Kralj et al., 2004a). The repetitions at the C-terminal region of glucansucrases have homology with motives for binding to bacterial cell wall present in choline-binding proteins, toxins, and other bacterial surface proteins (Leemhuis et al., 2013). However, the function of the C-terminal region is currently unknown, although its implication in several functions has been proposed: (i) polymerization or glucan structure, (ii) transfer of products to the catalytic center, and (iii) anchoring of the protein to the bacterial surface.

The *Lb. sakei* DsrLS and GTFKg15 enzymes only differ in the number of aa at their C-terminal (307 and 162 residues, respectively) and N-terminal (342 and 315 residues, respectively) regions, and the *Lb. reuteri* GTF180 lacks the C-terminal region.

These differences could be related to the enzymes processivity, since the dextrans synthesized in fermentation conditions by *Lb. sakei* MN1, *Lb. sakei* Kg15 and *Lb. reuteri* 180 have molecular masses of 1.7 × 10^8^ Da (Zarour et al., 2017), 2.7 × 10^7^ Da, and 3.6 × 10^6^ (Kralj et al., 2004a), respectively.

Here, we have demonstrated that the gene encoding DsrLS is carried by the 11,126 kbp pMN1 plasmid. Plasmidic localization of the genetic determinants for the production of EPS has been described previously (Wang and Lee, 1997). It has been determined, by plasmid curing, that the production of a HePS in *Lactobacillus casei* CG11 depends on the presence of a plasmid of 30 kbp (Kojic et al., 1992). Also, the *gtf* gene encoding the GTF glycosyltransferase, which synthesizes a *O*2-substituted (1,6)-β-D-glucan, has been identified in plasmids of *Pediococcus* and *Lactobacilli* strains (Werning et al., 2006). Concerning to dextran synthesis, it was shown that production of the polymer by two *Lactobacillus* strains isolated from meat was impaired upon curing of a 11 kbp plasmid (Ahrné et al., 1989), which could be identical or similar to pMN1. However, as far as we know, this is the first time that a plasmid carrying a gene encoding a dextransucrase has been completely sequenced.

Homology of pMN1 with other plasmids revealed that it belongs to a plasmid family whose prototype is pUCL287, which replicates *via* theta-mode. The pMN1 replicon includes two genes, *repA* and *repB*, which should encode the RepA and RepB proteins, involved in the initiation of plasmid replication and regulation of plasmid copy number. Thus, RepB could be responsible for the segregational stability of the plasmid and in fact the results obtained here indicate that low copy number pMN1 is stably inherited. In addition, the putative RelE toxin encoded by *orf4*, whose expression is probably regulated by the product of *orf3*, could be other mechanism to eliminate the bacterial population that has lost the plasmid.

**Figure 7:**
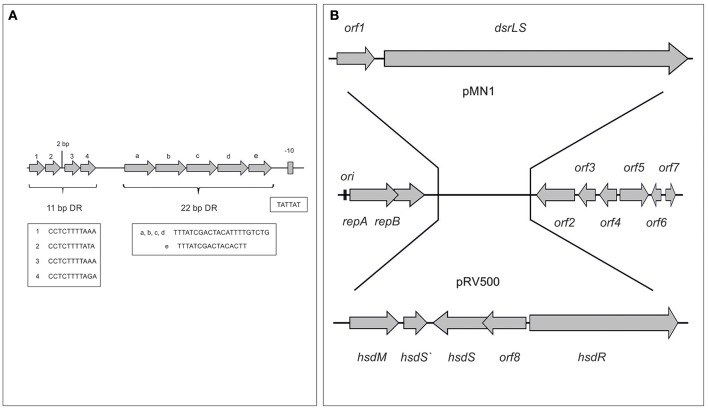
Origin of replication of pMN1 **(A)** and model of generation of pMN1 and pRV500 from a putative parental plasmid **(B)**. In **(A)** the sequences of the iterons and of the putative promoter of the *repA-repB-orf1-dsrLS* operon are depicted. In **(B)** the genes and the *orfs* of plasmids are depicted.

We have previously shown that *Lb. sakei* MN1 utilizes very efficiently sucrose with production of dextran and without accumulation of glucose, not affecting the growth rate and resulting in a higher biomass than when the growth medium was supplemented with glucose instead of sucrose (Nácher-Vázquez et al., 2017). Thus, it is not strange that the plasmid is segregationally stable because, in addition to encode RepB, it is not a burden for the cells and rather the bacteria is beneficiated by its presence. Upstream of *repA*, several iterons were identified: (i) 4 direct repeats of 11-bp (Figure 7A), which according to the studies performed with pUCL287 (Benachour et al., 1997) constitute the replication origin of the plasmid and presumably the RepA binding site, and (ii) 5 direct repeats of 22-bp, which could be involved in partitioning or incompatibility processes.

The BLAST analysis of the pMN1 DNA sequences vs. those deposited in the NCBI databases revealed homologies of ORF 2, 3, 4, 5, 6, and 7 with ORF 13, 12, 11, 10, 9, and 8 of the plasmid pRV500 from *Lb. sakei* RV332 (Alpert et al., 2003), which belongs to the same plasmid family as pMN1. The major difference between pRV500 and pMN1 is that the first one carries the genes encoding a restriction-modification type I system instead of the pMN1 *dsrLS* gene and its preceding *orf1*. This last one appears to be a truncated sequence of a gene that initially encoded a specific deoxyribonuclease (R protein), belonging to a type I restriction and modification system different to that of pRV500 (Figure 7B).

Most lactobacilli carry more than one plasmid and both pMN1 and pRV500 have been detected in *Lb. sakei* strains isolated from meat products, although in different countries. Thus, these plasmids seem to be derived from a parental plasmid composed of a replicon and the *orfs* 2–7, which subsequently by means of transposition processes incorporated modules that allow the synthesis of the dextran or a system of restriction and modification. Possibly, the acquisition of one or other module and its fixation is due to the selective advantage that supposes for their hosts against the environmental stress or the infection by bacteriophages. Moreover, the product of the *orf5*, a site-specific integrase could be involved in the process of modules exchange.

Depending on the requirement for sucrose utilization, expression of the dextransucrases could be constitutive or inducible. To date, it has been determined that its synthesis is constitutive in *Streptococcus* (Janda and Kuramitsu, 1978; Wenham et al., 1979), while it is inducible in presence of sucrose in *Leuconostoc* (Neely and Nott, 1962; Funane et al., 1995), although the molecular mechanisms of this induction are unknown. In *Weissella*, levels of dextransucrose detected in cultures of *Weissella cibaria* and *Weissella confusa* grown in presence of different sugars indicate constitutive expression of their Dsr enzymes (Bounaix et al., 2010). Determination of levels and activity of glucansucrases in *Lactobacillus* also points to a constitutive expression. This is the case of the glucan-producing *Lb.reuteri* TMW1106 (Schwab et al., 2007), the reuteran-producing *Lb. reuteri* 121 (Kralj et al., 2004a), and the dextran-producing *Lb. reuteri* 180 and *Lactobacillus parabuchneri* 33 (Kralj et al., 2004a).

In this work we have determined by RT-PCR analysis that the expression of the *dsrLS* gene of *Lb. sakei* MN1 did not increase when sucrose is present in the culture medium. Thus, the disaccharide is not an inducing agent of the *dsrLS* expression.

The transcriptional fusions generated in this work revealed two promoter regions designated P1 and P2, which drive expression of *dsrLS*. Inspection of the pMN1 sequences cloned in pRCR13 revealed a DNA sequence TATtAT (Figure 7A) which only deviates a nucleotide from the canonical −10 promoter sequence. This sequence is located 16 nucleotides upstream of *repA*, and could be P1. Also, within the pMN1 insert of pRCR15, a −10 extended promoter region (TGTTATtAT) with only one mismatch was observed 82 nucleotides upstream of *dsrLS*, that could correspond to P2. No −35 promoter region was detected for either P1 or P2. In addition, in late stationary phase cultures of *Lb. sakei* and not of *L. lactis* carrying pRCR15 a four-fold higher fluorescence levels was detected, when grown in medium containing only glucose (Tables 3, 4). Taking in consideration that *Lb. sakei* MN1 is the native background for the P2 promoter, it is feasible that a negative effector of the P2 promoter could be present or encoded by pMN1, pMN2 or the chromosome. Moreover, lower fluorescence was detected in *Lb. sakei* exponential cultures expressing mCherry from P1 or P2, when they were grown in MRSS. This fact could be due to the presence of dextran in cultures grown in MRSS, which could cause a shielding that masks fluorescence. However, analysis of individual cells by fluorescence microscopy also revealed a lower fluorescence in bacteria grown in MRSS medium (Figure 4), despite cells were washed repeatedly to remove the dextran prior to the analysis. In addition, difference in fluorescence levels between bacteria grown in MRSG or MRSS was not observed during exponential phase in MN1 strain when carried pRCR12 plasmid. Therefore, an alternative hypothesis is that the high synthesis of dextran (around 10 g L^−1^ in MRSS), although it does not seem to affect bacterial growth (Nácher-Vázquez et al., 2017), could finally be an energetic burden for the bacterium and the dextran itself could induce the activation of a inhibitory mechanism of its own synthesis at the transcriptional level. Another hypothesis, more feasible, is that the fructose synthesized as a consequence of the hydrolysis of sucrose, is the effector of inhibition. However, further studies will be necessary in order to elucidate the real cause of the detected effect.

An unexpected co-transcription of *dsrLS* with the *repA* and *repB* genes has been detected here. We believe that this is the first described case of co-transcription of genes involved in plasmid replication and a gene encoding a protein involved in the synthesis of EPS. However, this is not the first case in the literature of co-transcription of replication genes together with other genes, since the plasmidic *dysI* gene (encoding the immunity factor of the streptococcal bacteriocin dysgalacticin) is transcribed as part of the *copG*-*repB*-*dysI* replication-associated operon (Swe et al., 2010).

Finally, we would like to highlight the multicopy state of *dsrLS* in the pMN1 stable plasmid, its expression from two promoters not induced by sucrose, and the apparently processive DsrLS, which synthesizes a high-molecular mass dextran with antiviral and immunomodulatory activities (Nácher-Vázquez et al., 2017), as well as rheological properties (Zarour et al., 2017). Thus, all these facts support the potential of *Lb. sakei* MN1 and its dextran for multiple industrial applications including those in functional food.

## Author contributions

MN-V contributed to all parts of the experimental work and wrote a draft of the manuscript. JR-M performed the plasmid characterization. MM contributed to the transcriptional gene expression analysis. GdS performed the bioinformatics analysis of pMN1 plasmid, interpreted this analysis, and revised the manuscript. RA participated in study conception and corrected the manuscript. PL participated in study conception, data interpretation, and generated the final version of the manuscript. All authors have read and approved the final manuscript.

### Conflict of interest statement

The authors declare that the research was conducted in the absence of any commercial or financial relationships that could be construed as a potential conflict of interest.
