# Supplementary material for: Dextransucrase Expression Is Concomitant with that of Replication and Maintenance Functions of the pMN1 Plasmid in Lactobacillus sakei MN1
Source: Front Microbiol. 2017 Nov 21;8:2281. doi: 10.3389/fmicb.2017.02281 (PMC5702455; doi:10.3389/fmicb.2017.02281)
Supplement: Supplementary file 1 [file DataSheet1.PDF]

Supplementary material

Dextransucrase expression is concomitant with that of replication and maintenance functions of the pMN1 plasmid in *Lactobacillus sakei* MN1

Montserrat Nácher-Vázquez[1], José A. Ruiz-Masó[1], María L. Mohedano[1], Gloria del Solar G., Rosa Aznar[2,3] and Paloma López[1*].

[1]Biological Research Center, Department of Molecular Microbiology and Infection Biology, CSIC, Madrid, Spain.

[2]Institute of Agrochemistry and Food Technology,Department of Food Safety and Preservation, CSIC, Paterna, Spain.

[3]University of Valencia, Department of Microbiology and Ecology, Burjassot, Spain.

*** Correspondence:**

Paloma López
plg@cib.csic.es

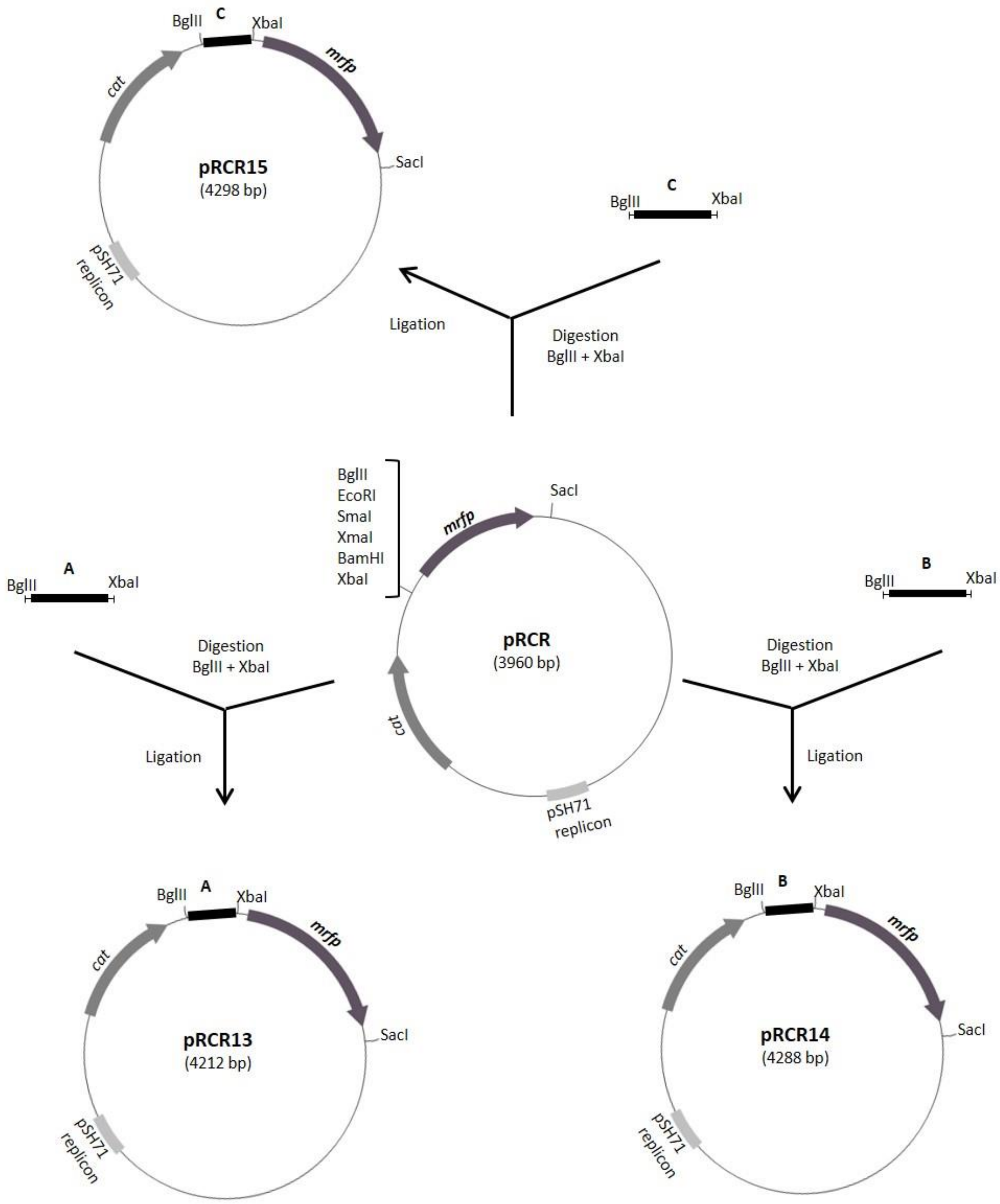

Figure S1. Scheme of construction of plasmids pRCR13, pRCR14 and pRCR15. Maps of these plasmids and of the parental pRCR are depicted. The pertinent restrictions sites as well as the *mrfp* gene encoding the mCherry and the *cat* gene, which encodes the chloramphenicol acetyltransferase, are shown.

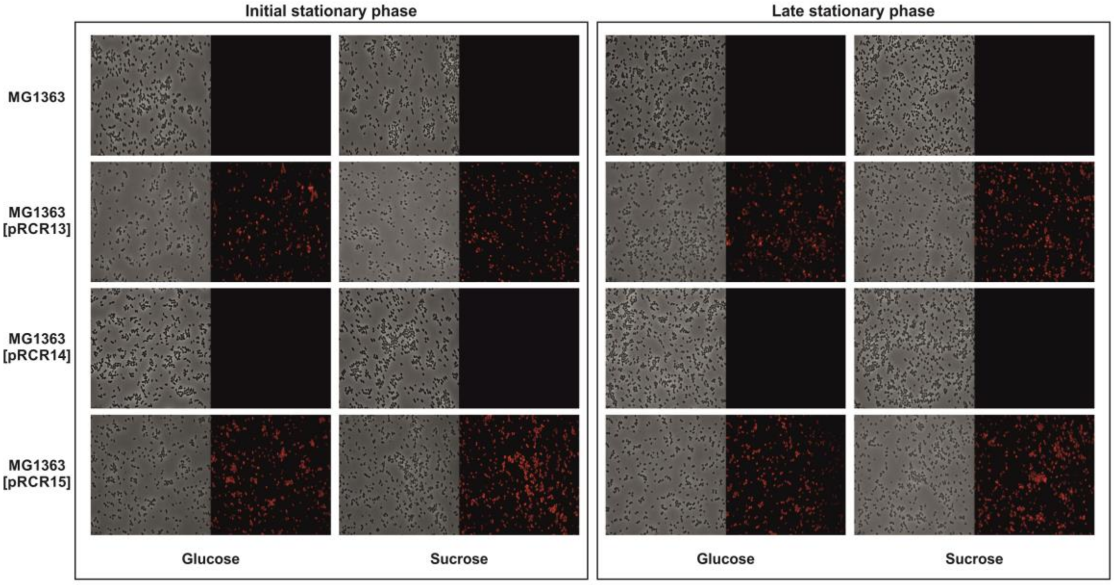

Figure S2. Detection of fluorescence in *Lactococcus lactis* strains. Cultures of the indicated strains grown in M17G (glucose) or M17GS (sucrose) and were analysed at the initial exponential phase or at late stationary phase by phase contrast (left panels) or fluorescence (right panels) microscopy.

Figure S3. Multiple alignment (CLUSTALX 2.1) of the amino acid sequences of the dextransucrases of *Lb. sakei* MN1, *Lb. sakei* Kg15 and *Lb. curvatus* 1624. Symbols: (*) identical amino acids in all sequences, (:), amino acids with very similar properties and (.), amino acids with properties with low similarity.

```
Lb. sakei MN1          MLRNNYFGETKTHYKLYKCGKNWAVMGISLFPLGLGMLVTSQPVSADVTATSTSSSAVRT 60
Lb. sakei Kg15         MLRNNYFGETKTHYKLYKCGKNWAVMGISLFPLGLGMLVTSQPVSADVTATSTSSSAVRT 60
Lb. curvatus TMW1624   MLRNNYFGETKTHYKLYKCGKNWAVMGISLFPLGLGMLVTSQPVSADVTATSTSSSAVRT 60
                       ************************************************************

Lb. sakei MN1          DAISESSSSAAKAETTSASSSSAVKAETTSASSSSAAKAETAAITTAGVANADSQTSAEV 120
Lb. sakei Kg15         DAIS--------------------------ASSSSAAKAETAAITTAGVANADSQTSAEV 94
Lb. curvatus TMW1624   DAISESSSSAAKAETTSASSSSAVKAETTSASSSSAAKAETAAITTAGVANADSQTSAEV 120
                       ****                          ******************************

Lb. sakei MN1          TADSTSTSQVVTNNSNNQNNTAQPAGQEAAPVSEDTSSDDSERTTPTVANNDKPAIDSVD 180
Lb. sakei Kg15         TADSTSTSQVVTNNSNNQNNTAQPAGQEAAPVSEDTSSDDSERTTPTVANNDKPAIDSVD 154
Lb. curvatus TMW162    TADSTSTSQVVTNNSNNQNNTAQPAGQEAAPVSEDTSSDDSERTTPTVANNDKPAIDSVD 180
                       ************************************************************

Lb. sakei MN1          TSQPATAAPKADTDVSTLQVDATTKTDSDIKEDTPTDKTTDTKTVQLTTVEGTSKQVVTT 240
Lb. sakei Kg15         TSQPATAAPKADTDVSTLQVDATTKTDSDIKEDTPTDKTTDTKTVQLTTVEGTSKQVVTT 214
Lb. curvatus TMW1624   TSQPATAAPKADTDVSTLQVDATTKTDSDIKEDTPTDKTTDTKTVQLTTVEGTSKQVVTT 240
                       ************************************************************

Lb. sakei MN1          PKEESSTDKSSSVVSKQTDKTSLPTVATATATTVSKIPSVTGDYQFDEKTKTYTFTGKDG 300
Lb. sakei Kg15         PKEESSTDKSSSVVSKQTDKTSLPTVATATATTVSKIPSVTGDYQFDEKTKTYTFTGKDG 274
Lb. curvatus TMW1624   PKEESSTDKSSSVVSKQTDKTSLPTVATATATTVSKIPSVTGDYQFDEKTKTYTFTGKDG 300
                       ************************************************************

Lb. sakei MN1          HPVTGLVYANNILQYFDETGHQVKGQYVTIAGHVYYFDPASGAAQTGVNQIDGKMVGFKS 360
Lb. sakei Kg15         HPVTGLVYANNILQYFDETGHQVKGQYVTIAGHVYYFDPASGAAQTGVNQIDGKMVGFKS 334
Lb. curvatus TMW1624   HPVTGLVYANNILQYFDETGHQVKGQYVTIAGHVYYFDPASGAAQTGVNQIDGKMVGFKS 360
                       ************************************************************

Lb. sakei MN1          DGSQITSGFSNDNAGNSYYFDESGTMVTGRQTIAGKTYYFDKDGHLRKGYSTIIDNQLYY 420
Lb. sakei Kg15         DGSQITSGFSNDNAGNSYYFDESGTMVTGWQTIAGKTYYFDKDGHLRKGYSTIIDNQLYY 394
Lb. curvatus TMW1624   DGSQITSGFSNDNAGNSYYFDESGTMVTGRQTIAGKTYYFDKDGHLRKGYSTIIDNQLYY 420
                       ***************************** ******************************

Lb. sakei MN1          FDLKTGESVSTTTSNFKSGLTSQTDDTTPHNSAVNMSKDSFTTVDGFLTAESWYVPKDIQ 480
Lb. sakei Kg15         FDLKTGESVSTTTSNFKSGLTSQTDDTTPHNSAVNMSKDSFTTVDGFLTAESWYVPKDIQ 454
Lb. curvatus TMW1624   FDLKTGESVSTTTSNFKSGLTSQTDDTTPHNSAVNMSKDSFTTVDGFLTAESWYVPKDIQ 480
                       ************************************************************

Lb. sakei MN1          TSATDWRASTPEDFRPIMMTWWPTKQIQAAYLNHMVSEGLLSSDKKFSATDDQTLLNQAA 540
Lb. sakei Kg15         TSATDWRASTPEDFRPIMMTWWPTKQIQAAYLNHMVSEGLLSSDKKFSATDDQTLLNQAA 514
Lb. curvatus TMW1624   TSATDWRASTPEDFRPIMMTWWPTKQIQAAYLNHMVSEGLLSSDKKFSATDDQTLLNQAA 540
                       ************************************************************

Lb. sakei MN1          HAVQLQIELKIQQTKSVEWLRTTMHNFIKSQPGYNVTSETPSNDHLQGGALSYINSVLTP 600
Lb. sakei Kg15         HAVQLQIELKIQQTKSVEWLRTTMHNFIKSQPGYNVTSETPSNDHLQGGALSYINSVLTP 574
Lb. curvatus TMW1624   HAVQLQIELKIQQTKSVEWLRTTMHNFIKSQPGYNVTSETPSNDHLQGGALSYINSVLTP 600
                       ************************************************************

Lb. sakei MN1          DANSNFRLMNRNPTQQDGTRHYNTDTSEGGYELLLANDVDNSNPVVQAEQLNWLYFLTHF 660
Lb. sakei Kg15         DANSNFRLMNRNPTQQDGTRHYNTDTSEGGYELLLANDVDNSNPVVQAEQLNWLYFLTHF 634
Lb. curvatus TMW1624   DANSNFRLMNRNPTQQDGTRHYNTDTSEGGYELLLANDVDNSNPVVQAEQLNWLYFLTHF 660
                       ************************************************************

Lb. sakei MN1          GEIVKNDPSANFDSVRVDAVDNVDADLLNITAAYFRDVYGVDKNDLTANQHLSILEDWGH 720
Lb. sakei Kg15         GEIVKNDPSANFDSVRVDAVDNVDADLLNITAAYFRDVYGVDKNDLTANQHLSILEDWGH 694
Lb. curvatus TMW1624   GEIVKNDPSANFDSVRVDAVDNVDADLLNITAAYFRDVYGVDKNDLTANQHLSILEDWGH 720
                          ************************************************************

Lb. sakei MN1          NDPLYVKDHGSDQLTMDDYMHTQLIWSLTKNPDNRSAMRRFMEYYLVDRAKDNTSDQAIP 780
Lb. sakei Kg15         NDPLYVKDHGSDQLTMDDYMHTQLIWSLTKNPDNRSAMRRFMEYYLVDRAKDNTSDPAIP 754
Lb. curvatus TMW1624   NDPLYVKDHGSDQLTMDDYMHTQLIWSLTKNPDNRSAMRRFMEYYLVDRAKDNTSDQAIP 780
                       ******************************************************** ***

Lb. sakei MN1          NYSFVRAHDSEVQTVIGDIVAKLYPDVKNSLAPSMEQLAAAFKVYDADMNSVNKKYTQYN 840
Lb. sakei Kg15         NYSFVRAHDSEVQTVIGDIVAKLYPDVKNSLP-SMEQLAAAFKVYDADMNSVNKKYTQYN 813
Lb. curvatus TMW1624   NYSFVRAHDSEVQTVIGDIVAKLYPDVKNSLAPSMEQLAAAFKVYDADMNSVNKKYTQYN 840
                       *******************************. ***************************
```

```
Lb. sakei MN1            MPAAYAMLLTNKDTIPRVYYGDMYTDDGQYMATKSPYYDAISALLKARIKYVAGGQTMAV 900
Lb. sakei Kg15           MPAAYAMLLTNKDTIPRVYYGDMYTDDGQYMATKSPYYDAISALLKARIKYVAGGQTMAV 873
Lb. curvatus TMW1624     MPAAYAMLLTNKDTIPRVYYGDMYTDDGQYMATKSPYYDAISALLKARIKYVAGGQTMAV 900
                         ************************************************************

Lb. sakei MN1            DKHDILTSVRFGDGIMNASDKGSTTARTQGIGVIVSNNDALALKGDTVTLHMGIAHANQA 960
Lb. sakei Kg15           DKHDILTSVRFGDGIMNASDKGSTTARTQGIGVIVSNNDALALKGDTVTLHMGIAHANQA 933
Lb. curvatus TMW1624     DKHDILTSVRFGDGIMNASDKGSTTARTQGIGVIVSNNDALALKGDTVTLHMGIAHANQA 960
                         ************************************************************

Lb. sakei MN1            YRALLLTTTDGLMKYTSDNGAPIRYTDANGDLIFTSADIKGYQNVEVSGFLSVWVPVGAS 1020
Lb. sakei Kg15           YRALLLTTTDGLMKYTSDNGAPIRYTDANGDLIFTSADIKGYQNVEVSGFLSVWVPVGAS 993
Lb. curvatus TMW1624     YRALLLTTTDGLMKYTSDNGAPIRYTDANGDLIFTSADIKGYQNVEVSGFLSVWVPVGAS 1020
                         ************************************************************

Lb. sakei MN1            DTQDARATGSSAANKTGDTLHSNAALDSNVIYEGFSNFQEMPTTHDEFTNVKIAQNADLF 1080
Lb. sakei Kg15           DTQDARATGSSAANKTGDTLHSNAALDSNVIYEGFSNFQEMPTAHDEFTNVKIAQNADLF 1053
Lb. curvatus TMW1624     DTQDARATGSSAANKTGDTLHSNAALDSNVIYEGFSNFQEMPTTHDEFTNVKIAQNADLF 1080
                         *******************************************:****************

Lb. sakei MN1            KSWGVTSFQLAPQYRSSDDTSFLDSIIKNGYAFTDRYDLGFNTPTKYGDVDDLADAIRAM 1140
Lb. sakei Kg15           KSWGVTSFQLAPQYRSSDDTSFLDSIIKNGYAFTDRYDLGFNTPTKYGDVDDLADAIRAM 1113
Lb. curvatus TMW1624     KSWGVTSFQLAPQYRSSDDTSFLDSIIKNGYAFTDRYDLGFNTPTKYGDVDDLADAIRAM 1140
                         ************************************************************

Lb. sakei MN1            HSVGIQVMADFVPDQIYNLPGQEVVAVNRTNNFGTPNQDSDLQNQLYVTNSKGGGEYQAK 1200
Lb. sakei Kg15           HSVGIQVMADFVPDQIYNLPGQEVVAVNRTNNFGTPNQDSDLQNQLYVTNSKGGGEYQAK 1173
Lb. curvatus TMW1624     HSVGIQVMADFVPDQIYNLPGQEVVAVNRTNNFGTPNQDSDLQNQLYVTNSKGGGEYQAK 1200
                         ************************************************************

Lb. sakei MN1            YGGEFLDLLRLEHPDLFTTNQISTGVPIDGSTKIKEWSAKYFNGSDIQGKGADYVLKDGA 1260
Lb. sakei Kg15           YGGEFLDLLRLEHPDLFTTNQISTGVPIDGSTKIKEWSAKYFNGSDIQGKGADYVLKDGA 1233
Lb. curvatus TMW1624     YGGEFLDLLRLEHPDLFTTNQISTGVPIDGSTKIKEWSAKYFNGSDIQGKGADYVLKDGA 1260
                         ************************************************************

Lb. sakei MN1            SQEYFKITSNANDESFLPKQFMNQDAMTGFTTDEKGTTYYSTSGYQAKQSFIQGDDGQYY 1320
Lb. sakei Kg15           SQEYFKITSNANDESFLPKQFMNQDAMTGFTTDEKGTTYYSTSGYQAKQSFIQGDDGQYY 1293
Lb. curvatus TMW1624     SQEYFKITSNANDESFLPKQFMNQDAMTGFTTDEKGTTYYSTSGYQAKQSFIQGDDGQYY 1320
                         ************************************************************

Lb. sakei MN1            YFDADGYMVTGSQTINGKQYYFLPNGVELREAFLQNASGNTVYYGKTGSAVKSKYVVDQS 1380
Lb. sakei Kg15           YFDADGYMVTGSQTINGKQYYFLPNGVELREAFLQNASGNTVYYGKTGSAVKSKYVVDQS 1353
Lb. curvatus TMW1624     YFDADGYMVTGSQTINGKQYYFLPNGVELREAFLQNASGNTVYYGKTGSAVKSKYVVDQS 1380
                         ************************************************************

Lb. sakei MN1            GVAYYFDVNGNMVADRMMILDGHTQYFFAGGSQAKDQFLIGSDGNLRYFDQGSGNMVTNR 1440
Lb. sakei Kg15           GVAYYFDVNGNMVADRMMILDGHTQYFFAGGSQAKDQFLIGSDGNLRYFDQGSGNMVTNR 1413
Lb. curvatus TMW1624     GVAYYFDVNGNMVADRMMILDGHTQYFFAGGSQAKDQFLIGSDGNLRYFDQGSGNMVTNR 1440
                         ************************************************************

Lb. sakei MN1            FAVNRNGDWFYFNGDGIALKGWQTIAGKTYFFDADGRQVKAAADKAAADKAAAEQAAADK 1500
Lb. sakei Kg15           FAVNRNGDWFYFNGDGIALKGWQTIAGKTYFFDADGRQVKAAAD---------------- 1457
Lb. curvatus TMW1624     FAVNRNGDWFYFNGDGIALKGWQTIAGKTYFFDADGRQVKAAAEQAAAEQAAADKAAADK 1500
                         *******************************************:

Lb. sakei MN1            AAADKAAAEQAAADKAAADKAAAEQAAADKAAADKAAAEQAAADKAAADKAAAEQAAAEQ 1560
Lb. sakei Kg15           ------------------------------------------------------------
Lb. curvatus TMW1624     AAAEQAAADKAAADKAAAEQAAAEQAAADKAAAEQAAADK-------------------- 1540

Lb. sakei MN1            AAADKAAAEQAAADKAAAEQAATDKAAADKAAAEQAAADKAAADKAAAEQAAAEQAAADK 1620
Lb. sakei Kg15           ------------------------------------------------------------
Lb. curvatus TMW1624     --------------------------------------------------AAAEQAAADK 1550

Lb. sakei MN1            AAAEQAAADKAAAEQAAADKAAAEQAAADKAAAKDKQTQAVAYAATKAKNNIDQATTADG 1680
Lb. sakei Kg15           ---------KAAAEQAAADKAAAEQAAADKAAAKDKQTQAVAYAATKAKNNIDQATTADG 1508
Lb. curvatus TMW1624     AAAEQAATDKAAADKAAAEQAAAEQAAADKAAAKDKQTQAVAYAATKAKNNIDQATTADG 1610
                                  ****::***::****************************************

Lb. sakei MN1            INDAQATGITDIDNQHVPGTSVDNQKQAEKVTEDIKNDPDNKTLPEAIELPNTGVDKTES 1740
Lb. sakei Kg15           INDAQATGITDIDNQHVPGTSVDNKKQAEKVTEDIKNDPDNKTLPEAIELPNTGVDKTES 1568
Lb. curvatus TMW1624     INDAQATGITDIDNQHVPGTSVDNQKQAEKVTEDIKNDPDNKTLPEAIELPNTGVDKTES 1670
                         ***********************:************************************
```

```
Lb. sakei MN1          ITITGVVMLILTTIFGLLFTSKKHKKD 1767
Lb. sakei Kg15         ITITGVVMLILTTIFGLLFTSKKHKKD 1595
Lb. curvatus TMW1624   ITITGVVMLILTTIFGLLFTSKKHKKD 1697
                       ***************************
```

Figure S4. Alignment (CLUSTALX 2.1) of the amino acid sequences of the dextransucrases of *Lb. sakei* MN1 (396-1395) and *Lb. reuteri* 180 3HZ3 (746-1751). Symbols: (*), identical amino acids in all sequences; (:), amino acids with very similar properties and (.), amino acids with low similarity.

```
Lb. sakei MN1         KTYYFD-KDGHLRKGYSTIIDNQLYYFDLKTGESVSTTTSNFKSGLTSQTDDTTPHNSAV 59
Lb. reuteri 180       -QYYIDPTTGQPRKNFLLQNGNDWIYFDKDTGAGTNALKLQFDKGTISADEQYRRGNEAY 59
                        **:* . *: **.:    .*:  *** .** ...: . :*..*  *  ::    *.*

Lb. sakei MN1         NMSKDSFTTVDGFLTAESWYVPKDIQTSATDWRASTPEDFRPIMMTWWPTKQIQAAYLNH 119
Lb. reuteri 180       SYDDKSIENVNGYLTADTWYRPKQILKDGTTWTDSKETDMRPILMVWWPNTVTQAYYLNY 119
                      . ...*: .*:*:***::** **:* ...* *  *.  *:***:*.***..  ** ***:

Lb. sakei MN1         MVSEG--LLSSDKKFSATDDQTLLNQAAHAVQLQIELKIQQTKSVEWLRTTMHNFIKSQP 177
Lb. reuteri 180       MKQYGNLLPASLPSFSTDADSAELNHYSELVQQNIEKRISETGSTDWLRTLMHEFVTKNS 179
                      * . *  * :*  .**:  *.: **: :. ** :** :*.:* *.:**** **:*:..:.

Lb. sakei MN1         GYNVTSETPSND--HLQGGALSYINSVLTPDANSNFRLMNRNPTQQDGTRHYNTDTSEGG 235
Lb. reuteri 180       MWNKDSENVDYGGLQLQGGFLKYVNSDLTKYANSDWRLMNRTATNIDGK-------NYGG 232
                       :*  **. . .  :**** *.*:** **  ***::*****..*: **.       . **

Lb. sakei MN1         YELLLANDVDNSNPVVQAEQLNWLYFLTHFGEIVKNDPSANFDSVRVDAVDNVDADLLNI 295
Lb. reuteri 180       AEFLLANDIDNSNPVVQAEELNWLYYLMNFGTITGNNPEANFDGIRVDAVDNVDVDLLSI 292
                       *:*****:*********:*****:* :** *. *:*.****.:*********.***.*

Lb. sakei MN1         TAAYFRDVYGVDKNDLTANQHLSILEDWGHNDPLYVKDHGSDQLTMDDYMHTQLIWSLTK 355
Lb. reuteri 180       ARDYFNAAYNMEQSDASANKHINILEDWGWDDPAYVNKIGNPQLTMDDRLRNAIMDTLSG 352
                      :  **. .*.:::.* :**:*:.****** :** **:. *. ****** ::. :: :*:

Lb. sakei MN1         NPDNRSAMRRFMEYYLVDRAKDNTSDQAIPNYSFVRAHDSEVQTVIGDIVAKLYPDVKNS 415
Lb. reuteri 180       APDKNQALNKLITQSLVNRANDNTENAVIPSYNFVRAHDSNAQDQIRQAIQAATGKPYGE 412
                       **:..*:.:::   **:**:***.: .**.*.*******:.*  * : :     .  ..

Lb. sakei MN1         LAPSMEQLAAAFKVYDADMNSVNKKYTQYNMPAAYAMLLTNKDTIPRVYYGDMYTDDGQY 475
Lb. reuteri 180       FN--LDDEKKGMEAYINDQNSTNKKWNLYNMPSAYTILLTNKDSVPRVYYGDLYQDGGQY 470
                      :   :::   .::.*  * **.***:. ****:**::******::*******:* *.***

Lb. sakei MN1         MATKSPYYDAISALLKARIKYVAGGQTMAVDKHDILTSVRFGDGIMNASDKGSTTARTQG 535
Lb. reuteri 180       MEHKTRYFDTITNLLKTRVKYVAGGQTMSVDKNGILTNVRFGKGAMNATDTGTDETRTEG 530
                      *  *: *:*:*: ***:*:*********:***:.***.****.* ***:*.*:  :**:*

Lb. sakei MN1         IGVIVSNNDALALK-GDTVTLHMGIAHANQAYRALLLTTTDGLMKYTSDNGAPIRYTDAN 594
Lb. reuteri 180       IGVVISNNTNLKLNDGESVVLHMGAAHKNQKYRAVILTTEDGVKNYTNDTDAPVAYTDAN 590
                      ***::***  * *: *::*.**** ** ** ***::*** **: :**.*..**: *****

Lb. sakei MN1         GDLIFTSADIKG--------YQNVEVSGFLSVWVPVGASDTQDARATGSSAANKTGDTLH 646
Lb. reuteri 180       GDLHFTNTNLDGQQYTAVRGYANPDVTGYLAVWVPAGAADDQDARTAPSDEAHTTKTAYR 650
                      *** **.:::.*        * * :*:*:*:****.**:* ****:: *. *:.*  : :

Lb. sakei MN1         SNAALDSNVIYEGFSNFQEMPTTHDEFTNVKIAQNADLFKSWGVTSFQLAPQYRSSDDTS 706
Lb. reuteri 180       SNAALDSNVIYEGFSNFIYWPTTESERTNVRIAQNADLFKSWGITTFELAPQYNSSKDGT 710
                      *****************   ***..* ***:***********:*:*:*****.**.* :

Lb. sakei MN1         FLDSIIKNGYAFTDRYDLGFNTPTKYGDVDDLADAIRAMHSVGIQVMADFVPDQIYNLPG 766
Lb. reuteri 180       FLDSIIDNGYAFTDRYDLGMSTPNKYGSDEDLRNALQALHKAGLQAIADWVPDQIYNLPG 770
                      ******.************:.**.***. :** :*::*:*..*:*.:**:**********

Lb. sakei MN1         QEVVAVNRTNNFGTPNQDSDLQNQLYVTNSKGGGEYQAKYGGEFLDLLRLEHPDLFTTNQ 826
Lb. reuteri 180       KEAVTVTRSDDHGTTWEVSPIKNVVYITNTIGGGEYQKKYGGEFLDTLQKEYPQLFSQVY 830
                      :*.*:*.*:::.**. : * ::* :*:**: ****** ******** *: *:*:**:

Lb. sakei MN1         ISTGVPIDGSTKIKEWSAKYFNGSDIQGKGADYVLKDGASQEYFKITSNANDESFLPKQF 886
Lb. reuteri 180       PVTQTTIDPSVKIKEWSAKYFNGTNILHRGAGYVLRSNDGKYYNLGTS---TQQFLPSQL 887
                        * ..** *.***********::*  :**.***:.. .: *   **    :.***.*:

Lb. sakei MN1         MNQD-AMTGFTTDEKGTTYYSTSGYQAKQSFIQGDDGQYYYFDADGYMVTG----SQTIN 941
Lb. reuteri 180       SVQDNEGYGFVKEGNNYHYYDENKQMVKDAFIQDSVGNWYYFDKNGNMVANQSPVEISSN 947
                        **    **..: :.  **. .    .*::***.. *::**** :* **:.    . : *

Lb. sakei MN1         GKQ--YYFLPNGVELREAFLQNASGNTVYYGKTGSAVKSKYVVDQSGVAYYFDVNGNMVA 999
Lb. reuteri 180       GASGTYLFLNNGTSFRSGLVKTDAG-TYYYDGDGRMVRNQTVSDG-AMTYVLDENGKLVS 1005
                      * .  * ** **..:*..:::. :* * **.  *  *:.: * *  .::* :* **::*:
```

Lb. sakei MN1	D 1000
Lb. reuteri 180	E 1006
	:
